# 2343: Enumeration of circulating tumor cells for monitoring cancer treatment response

**DOI:** 10.1017/cts.2017.223

**Published:** 2018-05-10

**Authors:** Jose Ignacio Varillas, Jinling Zhang, Weian Sheng, Kangfu Chen, Isis Barnes, Thomas George, Chen Liu, Hugh Fan

## Abstract

OBJECTIVES/SPECIFIC AIMS: The goal of this research is to use circulating tumor cells (CTC) enumeration and characterization to monitor anticancer treatment response. Emerging evidence strongly suggests the implications that epithelial-to-mesenchymal transition may have in cancer metastasis. Consequently, we hope to elucidate the significance of mesenchymal and stem-like CTCs in the peripheral blood of metastatic pancreatic cancer patients by analyzing the prevalence and frequency trends of CD133+ CTCs, as they relate to clinical events. We also hope to determine if there is a correlation between EpCAM+ CTCs and CD133+ CTCs numbers with tumor size, disease stage, and patient clinical outcome. METHODS/STUDY POPULATION: Blood samples of patients with metastatic pancreatic cancer (stage IV) were obtained from the University of Florida Health Cancer Center after informed consent through an IRB-approved protocol. CTC capture, characterization, and enumeration was performed on the blood of these cancer patients during their anticancer treatment. Patients had blood drawn for this purpose at time points aligned with clinical phlebotomy (every 2 weeks). CTC capture was performed by introducing treated patient blood samples into antibody-functionalized microdevices. The PDMS devices were functionalized by immobilizing either anti-EpCAM or anti-CD133, through an avidin-biotin complex. After capture, cells were fixated and permeabilized with 4% paraformaldehyde and 0.2% Triton X-100, respectively. Three-color immunocytochemistry (anti-cytokeratin-FITC, anti-CD45-PE, and DAPI) was performed to identify CTCs from nonspecifically captured blood cells. To be counted as a CTC, based on the FDA-approved technical definition, a cell with the appropriate cell size and morphology must be nucleated (DAPI+), express cytokeratin (CK+), and lack the leukocytic CD45 marker (CD45−). RESULTS/ANTICIPATED RESULTS: We tested the clinical utility of the device for monitoring the response of patients with advanced pancreatic cancer to a chemotherapy treatment consisting of anticancer drugs including 5-fluorouracil, leucovorin, oxaliplatin, and dasatinib. We have detected EpCAM+ CTCs in 47/47 (100%) and CD133+ CTCs in 41/47 (87.2%) of blood samples, coming from a cohort of 16 patients. We studied the correlation between the CTC numbers and the clinical result of patients in the study. We found that the size and changes in the size of the primary tumor (confirmed by CT scans) correlated with the frequency and increase/decrease trends in the number of CTCs detected. We expect to find some relationship between the number of detected CD133+ CTCs, or rather stem-like CTCs, and the clinical outcome of patients (eg, disease progression leading to withdrawal from study). DISCUSSION/SIGNIFICANCE OF IMPACT: Enumeration of patient CTCs and stem-like CTCs at different stages of a patient’s treatment may correlate with disease stage and prognosis, and prove useful in monitoring early recurrence, patient-specific treatment response, and newly acquired resistances; all of which would aid in providing guidance for the next step in clinical intervention. This type of liquid biopsy technology has great potential to make an impact in the future of personalized medicine and point-of-care diagnostics, as well as become a sturdy tool for translational research.

